# Design of a Measurement System for Six-Degree-of-Freedom Geometric Errors of a Linear Guide of a Machine Tool

**DOI:** 10.3390/s19010005

**Published:** 2018-12-20

**Authors:** Chien-Sheng Liu, Jia-Jun Lai, Yong-Tai Luo

**Affiliations:** 1 Department of Mechanical Engineering, National Cheng Kung University, Tainan City 70101, Taiwan; 2 Department of Mechanical Engineering, National Chung Cheng University, Chiayi County 62102, Taiwan; z22341068@gmail.com (J.-J.L.); aa0983426959@gmail.com (Y.-T.L.); 3 Advanced Institute of Manufacturing with High-tech Innovations, National Chung Cheng University, Chiayi County 62102, Taiwan

**Keywords:** geometric errors, skew-ray tracing, multiple-degree-of-freedom measurement, machine tool, position, straightness, pitch, roll, yaw, crosstalk

## Abstract

This paper proposes a system utilizing a Renishaw XL80 positioning error measuring interferometer and sensitivity analysis design to measure six-degree-of-freedom (6 DOF) geometric errors of a machine tool’s linear guide. Each error is characterized by high independence with significantly reduced crosstalk, and error calculations are extremely fast and accurate. Initially, the real light path was simulated using Zemax. Then, Matlab’s skew ray tracing method was used to perform mathematical modeling and ray matching. Each error’s sensitivity to the sensor was then analyzed, and curve fitting was used to simplify and speed up the mathematical model computations. Finally, Solidworks was used to design the set of system modules, bringing the proposed system closer to a product. This system measured actual 6 DOF geometric errors of a machine tool’s linear guide, and a comparison is made with the Renishaw XL-80 interferometer measurements. The resulting pitch, yaw, horizontal straightness, and vertical straightness error deviation ranges are ±0.5 arcsec, ±3.6 arcsec, ±2.1 μm, and ±2.3 μm, respectively. The maximum repeatability deviations for the measured guide’s pitch, yaw, roll, horizontal straightness, vertical straightness, and positioning errors are 0.4 arcsec, 0.2 arcsec, 4.2 arcsec, 1.5 μm, 0.3 μm, and 3 μm, respectively.

## 1. Introduction

With the advances of science and technology, the market demand for machine tools is increasing, and the finished products are becoming increasingly sophisticated and complex [1,2]. An essential part of a machine tool is the linear precision guide, which enables production of a good workpiece [3,4]. Due to machining errors and assembly errors, there are six-degree-of-freedom (6 DOF) geometric errors, including horizontal straightness error (*δx*), positioning error (*δy*), vertical straight line error (*δz*), pitch error (*εx*), roll error (*εy*), and yaw error (*εz*) [5,6]. Therefore, if a measurement system is implemented to check the errors of the motion guide, it will improve the accuracy and repeatability of the multi-axis machine tool, enable corresponding compensation afterwards, and improve the machining quality of the tool [7,8].

Measurement methods can be divided into two types: non-contact and contact. The non-contact type of measurement is most widely used, as in the long-stroke measurement method, for example. Moreover, a laser interferometer is often used in measuring the geometric error of the long-stroke linear precision guide of a machine tool [9,10,11]. The disadvantage of this method is that each measurement can only quantify a single geometric error. Measurement efficiency is notably low, and the measurement environment may change substantially, which makes guaranteeing accuracy of the measurement difficult during long measuring periods [4,8,12]. In recent years, various scholars have developed numerous methods to measure the 6 DOF geometric errors of linear precision guides [13,14,15,16,17,18,19,20,21,22,23]. For example, Sun et al. used Hall sensors and a permanent magnet (PM) composition measurement system to measure 3 DOF displacements [14]. Allred et al. proposed some novel applications and detailed sensitivity analysis of a 6 DOF displacement measuring device that used a Stewart platform structure [15]. A parallel mechanism was proposed by Mura et al. based on Stewart theory to measure the real deformation of a component [16,17,18].

Here, previously published methods relevant to this paper are detailed. Lee et al. proposed a 6 DOF geometric error measurement system for simultaneous measurement of a linear guide, but the route’s measured range was only 4 mm [7]. Kuang et al. proposed a 5 DOF geometric error linear guide measurement system for simultaneous measurement characterized by a large number of lenses or cymbals combined to simultaneously improve the sensitivity of each error [24]. Feng et al. proposed a system for simultaneously measuring 4 DOF geometric errors of a linear guide, which compensated for the drift of light [25]. A very simply structured system for simultaneously measuring 5 DOF geometric errors of a linear guide was proposed by Gao et al. [26]. Zhao et al. proposed another architecture for simultaneous measurement of 6 DOF geometric linear guide errors that combines a commercially available micro interferometer to measure the bit error and an optical fiber used for light source transmission [27]. The above literature indicated that there are limitations on simultaneous measurements of the long-stroke linear guide in terms of geometric optics, and it is difficult to maintain high accuracy and repeatability when measuring the positioning error, *ε*y, of the long stroke. The current solution is that the positioning error is assessed by means of interference. Our research group has been developing a multi-DOF geometric error measurement system. In 2016, we published a study that found that measurement distance is limited by a 3 DOF measurement system for a flat surface attitude [28], and we detailed its principles and verification method. We published a study of a 6 DOF measurement system for a linear guide in 2017 [4], in which the angle accuracy and straightness accuracy reached ± 1 μm and ± 0.2 arcsec, respectively, but the measurement was limited by the sensor size (only 6 mm). Subsequently, we proposed a modified 6 DOF measurement system for a linear guide in 2018, in which the measurable stroke could be up to 250 mm, and compensation for the light-induced drift was implemented [29]. 

Due to the special structure of the currently proposed measurement system design, the three light paths with three position sensitive detectors (PSDs) are highly independent, and the crosstalk can be reduced to a very low level in this system compared with the error in reference [29]. Unlike the roof prism used in reference [29], the proposed system uses a combination of a penta prism and a mirror, which has the advantage of reducing the crosstalk of the errors. When interpreting Figure 5 of reference [29], it is clear that the crosstalk situation is a critical consideration when all errors are introduced. Because the proposed measurement system of reference [29] used a Taylor series expansion in the analysis, the calculation time for each measurement was 5 minutes. However, the calculation time for this paper’s mathematical model is less than 1 second for each measurement in the proposed measurement system. The mathematical model refers to the mathematical formulas given herein by Equations (11)–(16). 

In this paper, we have proposed an improved 6 DOF geometric error measurement system for simultaneous measurement of long-stroke linear guides, which can measure a stroke of 500 mm with improved measurement accuracy, combined with a commercial interferometer Renishaw XL80 for measuring long-travel positioning error. In addition to measuring the commercially available linear guide on an optical table with the proposed prototype, the actual 6 DOF geometric errors of a machine tool’s linear guide were measured to assess the prototype’s effectiveness.

## 2. Structure Layout and Measuring Principle

**Figure 1:**
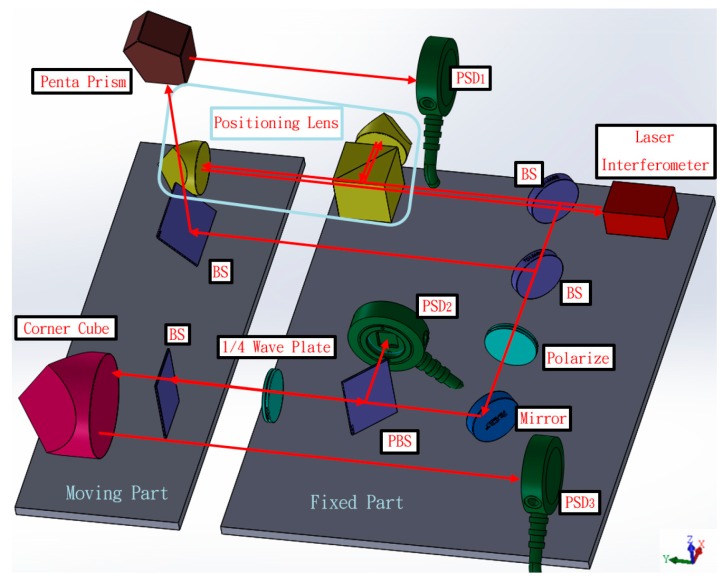
Structure of proposed measurement system.

The layout structure of the proposed measurement system for simultaneously measuring 6 DOF geometric errors of a long linear stage is shown in Figure 1. The framework consists of two parts: one fixed part and one moving part. Because the commercial interferometer Renishaw XL80 that is combined with this system (Figure 1) has a universal use in many laboratories, the laser source was replaced with a laser diode, and the positioning lens with the collocation error was excluded to simplify the experimental setup. Therefore, the simplified measurement system can only measure 5 DOF geometric errors, and therefore positioning error was measured using the commercial Renishaw XL80 interferometer. The remaining components of the fixed part include a beam splitter (BS), two mirrors, a polarizing beam splitter (PBS), a quarter wave plate (1/4 Wave Plate), a polarizer, and three position sensitive detectors (PSDs) for receiving the light source. The moving portion includes two BSs, a pentagonal edge penta prism, and a corner cube. The fixed part of the system module was attached outside the measured linear guide in order to emit the measuring laser beam and receive the position signal of the laser’s light. Furthermore, the moving part was placed on the measured linear guide. The laser light therefore hits the moving part and returns PSDs toward the fixed part. Through the movement of the linear guide, the laser light will be deflected, and the PSDs can read the location changes and utilize the change values in the mathematical model. Finally, the 6 DOF geometric errors of the measured linear guide can be calculated.

**Figure 2:**
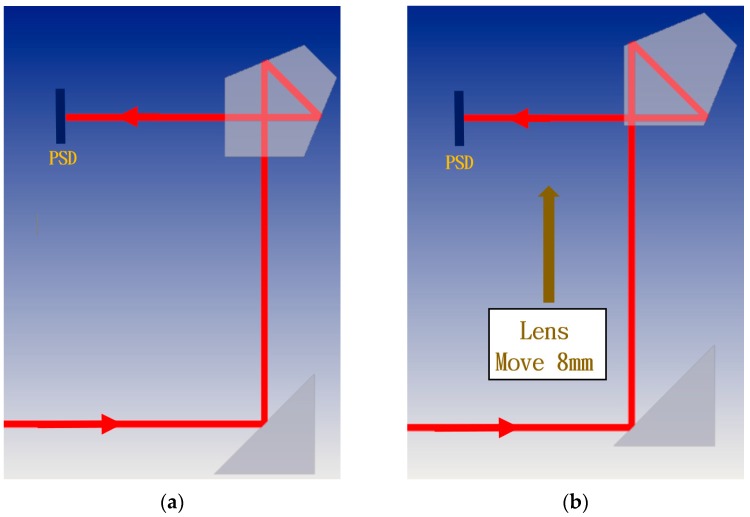
Light path with mirror and penta prism: (**a**) before and (**b**) after mirror’s movement.

**Figure 3:**
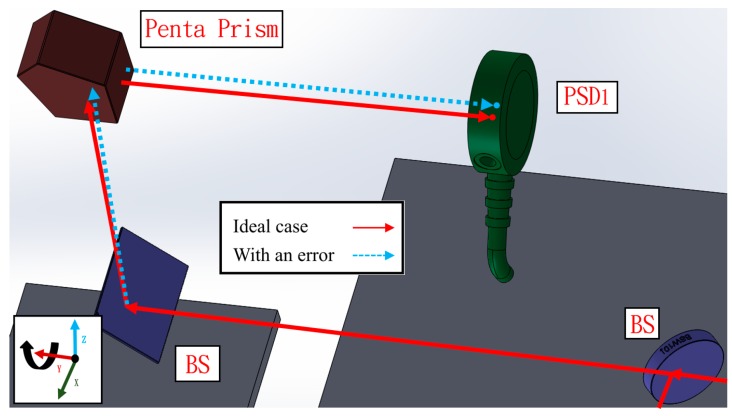
Light path with roll error.

The proposed measurement system can be divided into three light paths with three PSDs. The measurement principle is introduced separately in the following section. In the first light path, the laser beam passes through the BS and penta prism of the moving part. The straightness errors in the X and Z directions have extremely low sensitivity to PSD 1, and thus the error crosstalk can be reduced. Additionally, angle errors can be accurately obtained by PSD 1. Figure 2a,b show the light path with a mirror and a penta prism before and after the mirror’s movement, respectively. As shown in Figure 2b, when the mirror moves 8 mm toward the penta prism (i.e., the straightness error is 8 mm), the light spot position on PSD 1 changes slightly. When the linear guide with 6 DOF geometric errors moves to different positions, the optical paths of the laser beams in the proposed measurement system change, and consequently the positions of the light spots on the PSDs also change. Figure 3 shows the change in the light path when there is a roll error (*εy*).

**Figure 4:**
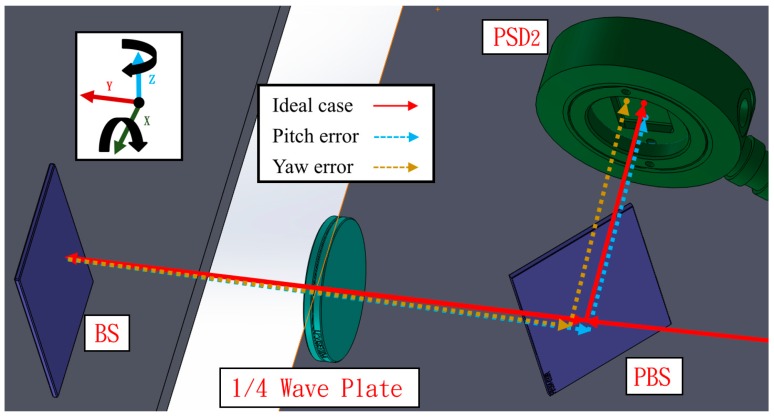
Light path with pitch error or yaw error.

In the second light path, the laser beam passes through the BS on the moving part and returns to the S-polarized light by rotating the original P-polarized light by 90° through the quarter wave plate, and subsequently reflecting from the PBS to PSD 2. This design can reduce the number of components used and the error crosstalk. Except for pitch and yaw errors, the remaining errors hardly impact PSD 2, which improves the measurement accuracy. Figure 4 shows the variation in the light path when a pitch error (*εx*) or yaw error (*εz*) occurs. 

**Figure 5:**
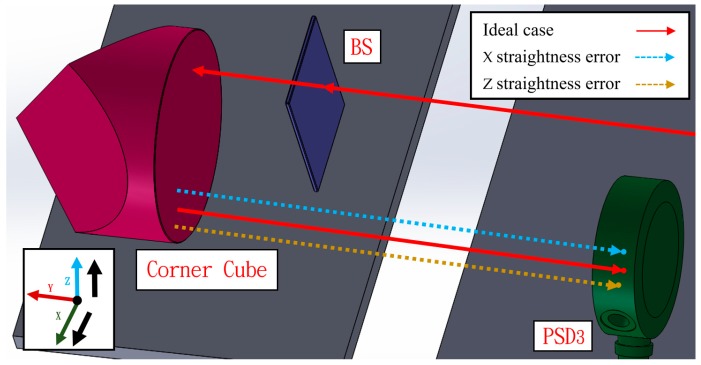
Light path with horizontal straightness error or vertical straightness error.

In the third light path, the laser beam passes through the BS and the corner cube. Uniquely, the corner cube can magnify the straightness error change by a factor of two. Furthermore, the sensitivity to other errors besides the straightness error and roll error is low, which can reduce the error crosstalk and allow the beam to be reflected 180 degrees using only one component. Figure 5 shows the variation in the light path when there is a horizontal straightness error (*δx*) or a vertical straightness error (*δz*).

## 3. Numerical Simulation and Mathematical Model

**Figure 6:**
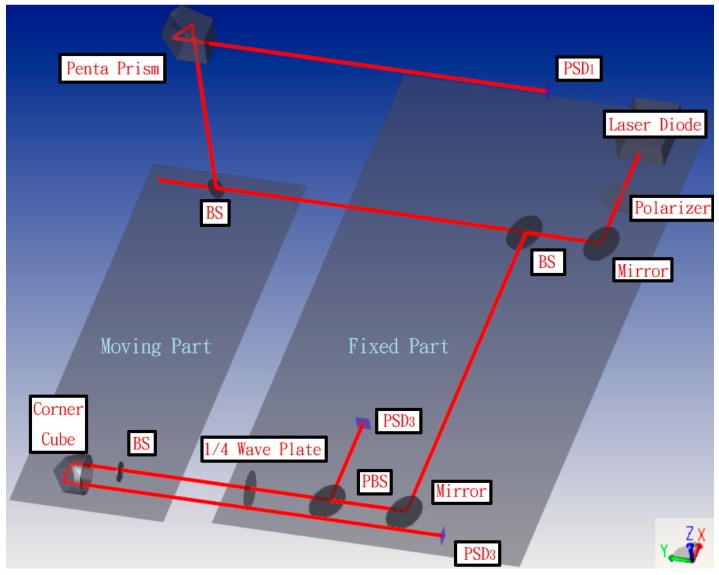
Zemax 3D optical model of the proposed measurement system.

**Figure 7:**
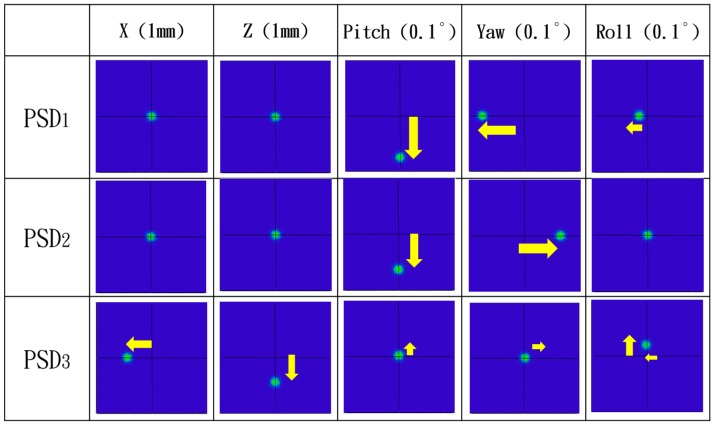
Simulation results showing variation of light spot positions on PSDs with geometric errors.

In this paper, the ray trace function of the Zemax software was used to verify the measurement performance of the proposed system and simulate the positions of the light spots on the PSDs qualitatively. Figure 6 shows the Zemax 3D optical model of the proposed measurement system. Figure 7 illustrates the optical simulation results obtained for the changes in the laser spot locations on the PSDs under different error conditions with respect to horizontal straightness, vertical straightness, pitch, yaw, and roll. It is notable that numerical simulation of the positioning error is omitted, because the measurement of the positioning error was carried out using the commercial laser interferometer. These optical simulation results suggest a high feasibility for utilizing the proposed measurement system. These spot changes are also the calculation basis for our mathematical model. We will ignore the low-sensitivity spot changes and retain the high-sensitivity spot changes in order to solve for all errors with a high response.

**Figure 8:**
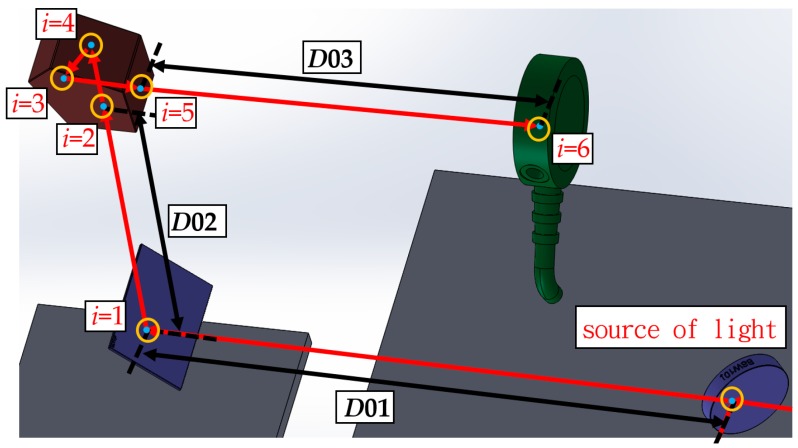
Example boundary surfaces of proposed measurement system.

Next, the mathematical model derivation is briefly introduced along with its characteristics. Further information about the mathematical model can be found in our previous papers [29,30,31,32,33]. Our mathematical models are built using the skew-ray tracing method and a homogeneous transformation matrix (HTM). In the example shown in Figure 8, we use the HTM to define the boundaries of each optical component relative to our frame of reference. 

Following the algorithm for the flat-boundary skew-ray tracing method, shown in our previous publication [29,30,31,32,33], AiR denotes the transfer matrix from each optical device (i) coordinate system to the reference coordinate system (R), and is given as:

(1) AiR=[IixIiyIiz0JixJiyJiz0KixKiyKiz0tixtiytiz1]

As shown in Figure 8, the incident point on the surface of the *i*th optical component can be represented as $P_{i-1}={\left[\begin{array}{cccc} P_{i-1x} & P_{i-1y} & P_{i-1z} & 1 \end{array}\right]}^{T}$, and the unit directional vector of the laser beam is expressed as $\ell_{i-1}={\left[\begin{array}{cccc} \ell_{i-1x} & \ell_{i-1y} & \ell_{i-1z} & 0 \end{array}\right]}^{T}$. When the ray impacts the flat surface, if $\lambda_{i}$ is the vector from the source $P_{i-1}$ to the destination point $P_{i}$, and $\lambda_{i}$ is determined as follows:

(2) $$P_{i}={\left[\begin{array}{cccc} P_{ix} & P_{iy} & P_{iz} & 1 \end{array}\right]}^{T}={\left[\begin{array}{cccc} P_{i-1x}+\ell_{i-1x}\lambda_{i} & P_{i-1y}+\ell_{i-1y}\lambda_{i} & P_{i-1z}+\ell_{i-1z}\lambda_{i} & 1 \end{array}\right]}^{T},$$

(3) $$\lambda_{i}=\frac{-(I_{iz}P_{i-1x}+J_{iz}P_{i-1y}+K_{iz}P_{i-1z}+t_{iz})}{I_{iz}\ell_{i-1x}+J_{iz}\ell_{i-1y}+K_{iz}\ell_{i-1z}}=\frac{-B_{i}}{G_{i}}$$

According to Snell’s Law, the unit directional vector, $\ell_{i}$, of the reflected ray is as follows:

(4) $$\ell_{i}={\left[\begin{array}{cccc} \ell_{ix} & \ell_{iy} & \ell_{iz} & 0 \end{array}\right]}^{T}={\left[\begin{array}{cccc} \ell_{i-1x}-2I_{iz}G_{i} & \ell_{i-1y}-2J_{iz}G_{i} & \ell_{i-1z}-2K_{iz}G_{i} & 0 \end{array}\right]}^{T}.$$

We can calculate the unit directional vector of reflected or refracted light on one plane whenever the beam hits the subsequent plane, beginning from the origin of the laser. Using this method, we can trace the laser path through the system continuously, and therefore we can obtain the image centroid coordinates of the light spots on the PSDs as follows:

*X*_PSD1_ = *F_X_*_1_(*δ_x_*, *δ_z_*, *ε_x_*, *ε_y_*, *ε_z_*), (5)

*Y*_PSD1_ = *F_Y_*_1_(*δ_x_*, *δ_z_*, *ε_x_*, *ε_y_*, *ε_z_*), (6)

*X*_PSD2_ = *F_X2_*(*δ_x_*, *δ_z_*, *ε_x_*, *ε_y_*, *ε_z_*), (7)

*Y*_PSD2_ = *F_Y2_*(*δ_x_*, *δ_z_*, *ε_x_*, *ε_y_*, *ε_z_*), (8)

*X*_PSD3_ = *F_X3_*(*δ_x_*, *δ_z_*, *ε_x_*, *ε_y_*, *ε_z_*), (9)

*Y*_PSD3_ = *F_Y3_*(*δ_x_*, *δ_z_*, *ε_x_*, *ε_y_*, *ε_z_*) (10)

where X_PSD*i*_ (*i* = 1, 2, and 3) and Y_PSD*i*_ (*i* = 1, 2, and 3) are the image centroid coordinates of the light spot on PSD*i* in the X-direction and Y-direction, respectively. 

Next, we analyze the contribution of each error to the change of the sensor readings in order to remove the low-sensitivity errors and simplify the mathematical model, thus improving the calculation speed without decreasing accuracy. The technique requires differentiating individual equations for individual errors, and then assessing the contribution of the error to the equation. Finally, with the curve fitting method, the mathematical model can be simplified into a clear formula as follows:

*X*_PSD1_ = *Aεy*+*Bεz*+*C*, (11)

*Y*_PSD1_ = *Dεx*+*E*, (12)

*X*_PSD2_ = *Fεz*+*G*, (13)

*Y*_PSD2_ = *Hεx*+*I*, (14)

*X*_PSD3_ = *Jδx*+*Kεy*+*Lεz*+*M*, (15)

*Y*_PSD3_ = *Nδz*+*Oεy*+*Pεx+Q*, (16)

where, A–Q are known constants. 

**Figure 9:**
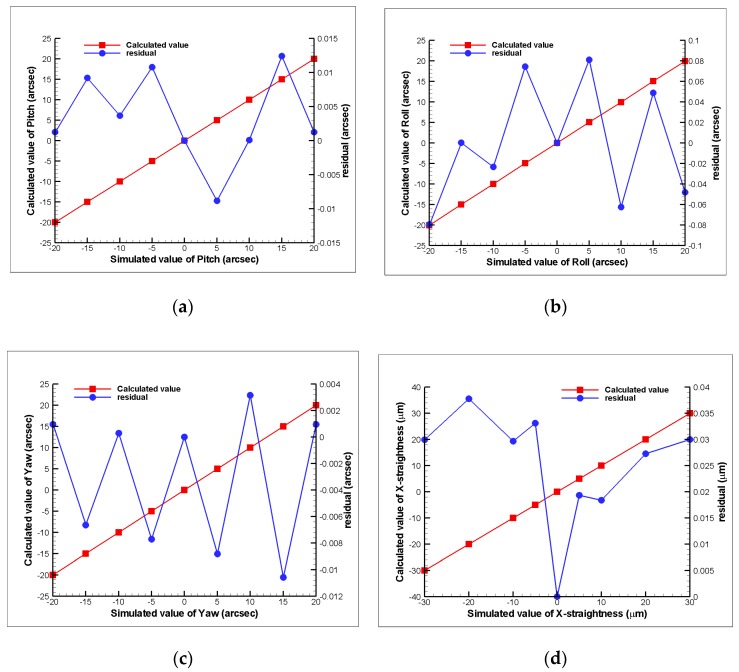

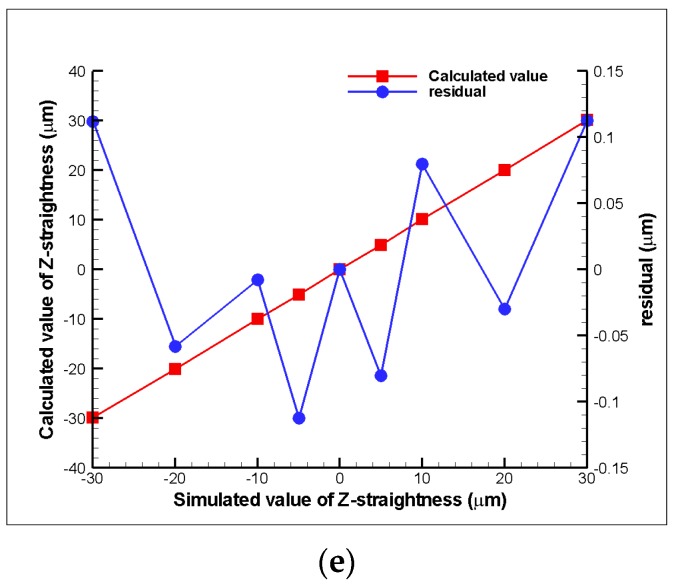
Verification of the mathematical model: (**a**) pitch, (**b**) roll, (**c**) yaw, (**d**) horizontal straightness, and (**e**) vertical straightness, respectively.

After Zemax simulation of the proposed measurement system with the compound errors, the obtained changes in location of the light spots on the PSDs were utilized in the proposed mathematical model to obtain the calculated errors. Figure 9 shows the comparison between the calculated results from the mathematical model and the Zemax simulation results. In the figures, the residual values are those of the proposed measurement system results and are small enough for machine tool applications.

## 4. Experimental characterization

**Figure 10:**
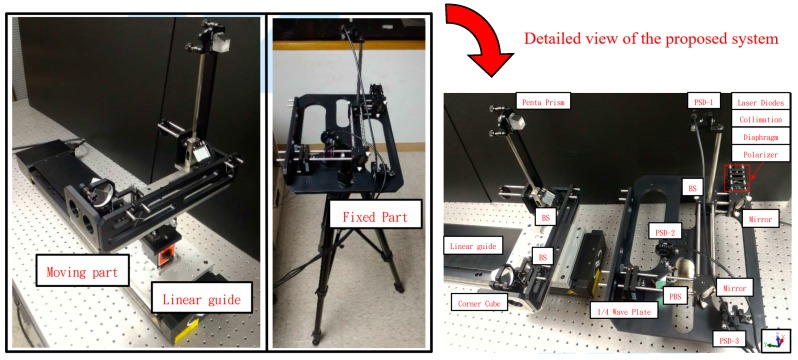
Photograph of laboratory-built prototype.

**Figure 11:**
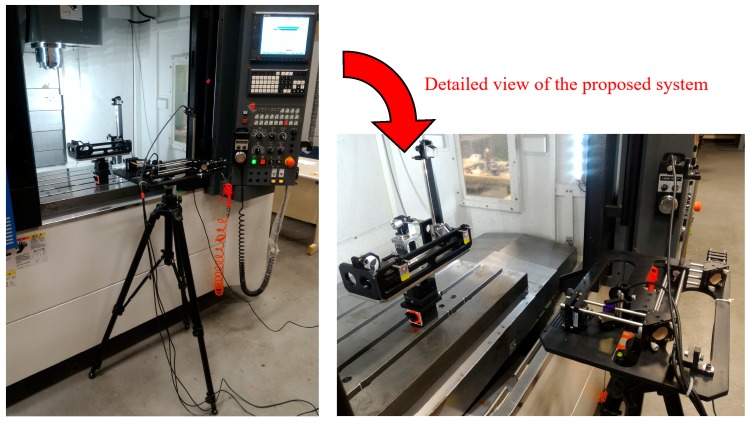
Measured machine tool and proposed prototype.

**Figure 12:**
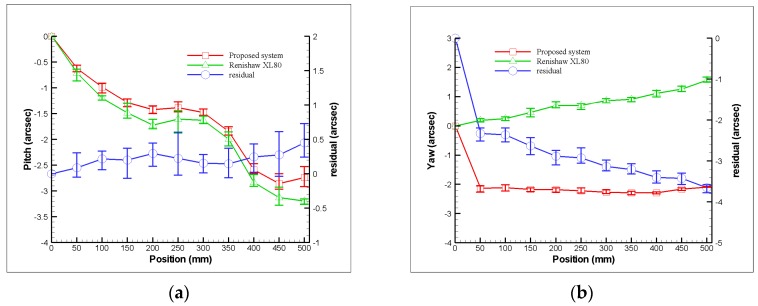

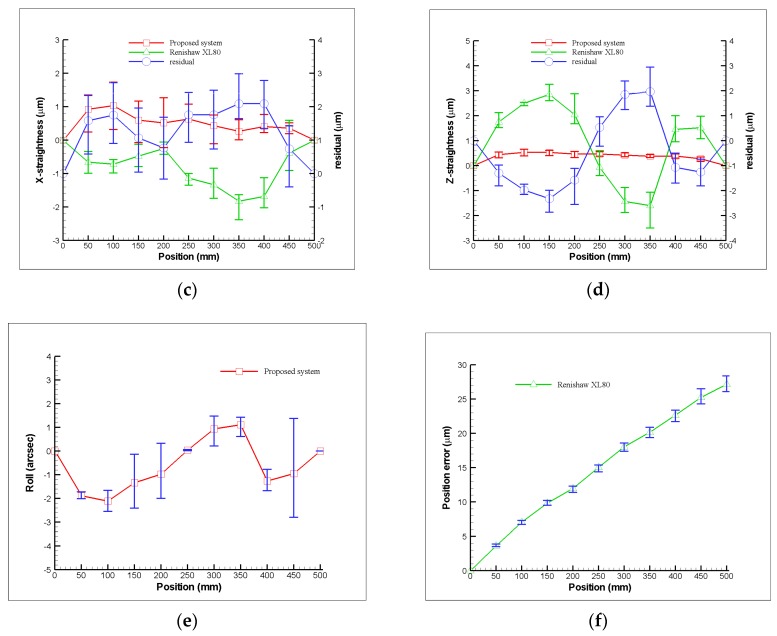
Measurement results for variation of geometric error with position: (**a**) pitch, (**b**) yaw, (**c**) horizontal straightness, (**d**) vertical straightness, (**e**) roll, and (**f**) positioning errors, respectively.

As shown in Figure 10 and Figure 11, the Renishaw XL-80 interferometer was replaced with a diode laser in the experimental setup for simultaneous measurement of the horizontal straightness, vertical straightness, pitch, yaw, and roll errors. In the experiment, the prototype of the proposed measurement system was used to simultaneously measure the 5 DOF geometric errors of the linear guide of a three-axis machine tool (VC-608, Tongtai, Kaohsiung, Taiwan) in the factory. Subsequently, the Renishaw XL-80 interferometer was used to measure the individual geometric errors of the linear guide and verify the performance of the proposed measurement system. It is noteworthy that the Renishaw XL-80 interferometer can only quantify a single geometric error at each measurement, and it cannot measure the roll error. In the experiment, the linear guide was controlled to move 50 mm between measurements, and the ambient temperature was set. The linear guide can be moved directly via a linear motor, which features a closed-loop control scheme based upon a feedback signal generated with an optical encoder (having a repeatability of 2.64 μm). The right side of Figure 10 shows a detailed view of the proposed measurement system, in which it clearly appears to be an existing preliminary module design. Figure 12 shows a series of measurement results with 5 repeated measurements at each position in comparison to those of the Renishaw XL80 interferometer. The measurement results show that the deviation ranges for the pitch, yaw, horizontal straightness, and vertical straightness errors are ±0.5 arcsec, ±3.6 arcsec; ±2.1 μm, and ±2.3 μm, respectively. For the measured linear guide, the maximum deviations in repeatability for pitch, yaw, roll, horizontal straightness, vertical straightness, and positioning errors are 0.4 arcsec, 0.2 arcsec, 4.2 arcsec, 1.5 μm, 0.3 μm, and 3 μm, respectively. However, it is important to note that the roll error cannot be measured using the interferometer, while the positioning error is measured directly by the interferometer. The above results demonstrate that the proposed system is capable of measuring the 6 DOF geometric errors of the long-stroke linear guide. However, Abbe error and the laser disturbance cause imperfections in the measured results. Therefore, to improve the system’s measurement accuracy, these issues must be considered, and the system further optimized in the future [34,35,36,37].

## 5. Conclusions

This paper proposes a new type of non-contact optical measurement system for simultaneously measuring 6 DOF geometric errors of a linear guide of a machine tool. In the studied method, a commercial Renishaw XL80 interferometer was combined to the newly proposed measurement system to measure the long-stroke positioning error (*δy*). The experimental results indicate that the proposed measurement system can simultaneously measure the linear guide’s 6 DOF geometric errors with a stroke of 500 mm. For the measured linear guide, the maximum deviations in the repeatability of pitch, yaw, roll, horizontal straightness, vertical straightness, and positioning errors are 0.4 arcsec, 0.2 arcsec, 4.2 arcsec, 1.5 μm, 0.3 μm, and 3 μm, respectively. When compared with the Renishaw XL80 interferometer, the proposed measurement system has a greatly reduced measuring time and lowered cost due to its simple structure.

## 6. Patents

The authors also published a Taiwan patent I614513 resulting from the work reported in this manuscript.
